# Nanocomposites Based on Luminescent Colloidal Nanocrystals and Polymeric Ionic Liquids towards Optoelectronic Applications

**DOI:** 10.3390/ma7010591

**Published:** 2014-01-21

**Authors:** Annamaria Panniello, Chiara Ingrosso, Paul Coupillaud, Michela Tamborra, Enrico Binetti, Maria Lucia Curri, Angela Agostiano, Daniel Taton, Marinella Striccoli

**Affiliations:** 1 Institute for Physical and Chemical Processes (IPCF), Bari Division, National Research Council C.N.R. c/o, Department of Chemistry, via Orabona 4, 70126 Bari, Italy; E-Mails: a.panniello@ba.ipcf.cnr.it (A.P.); c.ingrosso@ba.ipcf.cnr.it (C.I.); m.tamborra@ba.ipcf.cnr.it (M.T.); enrico.binetti@unitn.it (E.B.); lucia.curri@ba.ipcf.cnr.it (M.L.C.);; 2 Laboratoire de Chimie des Polymères Organiques, Centre National de la Recherche Scientifique and Laboratoire de Chimie des Polymères Organiques, Université Bordeaux, Ecole Nationale Supérieure de Chimie de Biologie and de Physique, 16, Avenue Pey-Berland, 33607 Pessac Cedex, France; E-Mails: paul.coupillaud@enscbp.fr (P.C.); taton@enscbp.fr (D.T.); 3 Now at Institute for Composite and Biomedical Materials, National Research Council, Via Sommarive, 14, 38123 Trento, Italy; 4 Department of Chemistry, University of Bari “Aldo Moro”, Via Orabona 4, 70126 Bari, Italy; E-Mail: angela.agostiano@uniba.it (A.A.)

**Keywords:** colloidal nanocrystals, polymeric ionic liquids, nanocomposites, surface functionalization, time-resolved spectroscopy

## Abstract

Polymeric ionic liquids (PILs) are an interesting class of polyelectrolytes, merging peculiar physical-chemical features of ionic liquids with the flexibility, mechanical stability and processability typical of polymers. The combination of PILs with colloidal semiconducting nanocrystals leads to novel nanocomposite materials with high potential for batteries and solar cells. We report the synthesis and properties of a hybrid nanocomposite made of colloidal luminescent CdSe nanocrystals incorporated in a novel *ex situ* synthesized imidazolium-based PIL, namely, either a poly(*N*-vinyl-3-butylimidazolium hexafluorophosphate) or a homologous PIL functionalized with a thiol end-group exhibiting a chemical affinity with the nanocrystal surface. A capping exchange procedure has been implemented for replacing the pristine organic capping molecules of the colloidal CdSe nanocrystals with inorganic chalcogenide ions, aiming to disperse the nano-objects in the PILs, by using a common polar solvent. The as-prepared nanocomposites have been studied by TEM investigation, UV-Vis, steady-state and time resolved photoluminescence spectroscopy for elucidating the effects of the PIL functionalization on the morphological and optical properties of the nanocomposites.

## 1. Introduction

In the few last years, nanoscience has attracted the interest of the scientific community thanks to the emerging and enormous advantages offered by materials at the nanoscale for applications in numerous technological fields, such as photonics, electro-optics, magnetic storage, sensing, catalysis, biotechnologies and energy conversion and storage [1]. Among the various preparation methods, colloidal routes represent an effective and valuable tool for obtaining nanoparticles (NPs) and nanocrystals (NCs) in a wide range of compositions, shapes and dimensions [2]. The surface of colloidal NCs is typically capped with a layer of ligand molecules with long hydrocarbon tails, which allow for their dispersion in weakly polar media and introduce an insulating layer around each nano-object [2]. As a result, for practical applications, the advantages of quantum-confined materials are often balanced by ineffective injection or extraction of electrons and holes, for example in the production of light emitting diodes (LEDs) or other energy devices. The use of small inorganic ligands instead of traditional capping molecules with long hydrocarbon tails has been demonstrated to facilitate the charge transport between individual NCs, opening up interesting opportunities for their integration in devices. Talapin *et al.* [3] have reported the use of inorganic molecular metal chalcogenide (MCC) ligands that provide colloidal stabilization without blocking the interparticle charge transport. The MCC ligands contain main group or transition metals, which can bind the NC surface through chalcogenide bridges. Nevertheless, the presence of transition metal ions in close proximity to the NC surface could, in some cases, detrimentally affect the chemical and physical properties of the nano-objects, for example participating in red-ox processes. In addition, MCCs are typically synthesized and then processed in a highly toxic and pyrophoric hydrazine medium, with great caution under an inert environment, limiting the scale-up and application of this approach.

As a matter of fact, new approaches and novel functionalities are currently under investigation for designing capping ligands capable of favoring charge transport among NCs when integrated in electronic devices. Recently, Talapin *et al.* [3] have explored new capping molecules as metal-free inorganic ligands for achieving all-inorganic colloidal NCs, such as chalcogenides, hydrochalcogenides and mixed chalcogenides. These ligands are the simplest, cheapest and smallest ones for colloidal NCs, resulting, at the same time, in safe handling; thus, they can find a broad use in NC-based devices [4]. Moreover, these inorganic ions bind to the surface of semiconductor and metal NCs and provide electrostatic stabilization for colloidal dispersion in polar solvents.

The opportunity for modifying the NC surface by such inorganic ligands provides stabilization of NCs in different polar environments, thus extending the range of materials with which the NCs can interact and improving the practical applications of the nano-objects. A broad diversity of host media with defined physical, chemical and mechanical properties has been exploited to produce nanocomposites formed of colloidal NCs. Besides polymers, liquid crystals and small organic molecules, also ionic liquids (ILs) have been recently used as a host matrix to transfer the peculiar properties of inorganic NPs in a unique nanocomposite material. ILs have attracted interest because of their high ionic conductivity, wide electrochemical window and negligible vapor pressure [5].

The opportunity to conjugate the peculiar properties of ILs with the specific features of polymeric materials represents an additional resource to the current research activities in the energy storage and conversion field [6–11]. Indeed, such a combination improves the IL potential, with the easy processability of polymers, improving mechanical stability and dimensional control, thus offering new outlooks and exciting challenges towards new applications [6]. In the last decade, polymeric ionic liquids (PILs) have been joining to the range of functional materials for numerous practical applications, such as the design and fabrication of solid-state polymer electrolytes in lithium-polymer batteries [12,13], dye-sensitized solar cells [14], active materials for the production of optically transparent and ion-conductive polymer coatings [15] and multifunctional electro-optic actuators [16]. Recently, the development of controlled/living radical polymerization techniques, such as atom transfer radical polymerization (ATRP) [17] and reversible addition-fragmentation chain transfer (RAFT) [18], has dominated the field of polymer synthesis. The opportunity to precisely design and control polymer architecture by such techniques has been applied also in integrating IL species on a meso-/nano-scale in a polymer matrix. Such polymerization methods have been successfully exploited to prepare homopolymers with a well-defined structure, as well as PIL-based block copolymers.

Growing attention has recently aimed at integrating inorganic nanostructures in PILs for numerous applications. For instance, Nakashima *et al*. [19,20] have reported the preparation of nanocomposites formed by luminescent semiconducting quantum dots (QDs) and PILs. Moreover, ordered structures have been also obtained by assembling various types of NPs in ordered ionic polymeric structures derived from ionic liquids [21]. PILs have also been exploited as a dispersant medium for the stabilization of Au nanorods to obtain composite materials that are environmentally responsive and that can be used in self-assembling [22], rhodium NPs as effective catalysts [23] or carbon nanotubes as electroconductive soft materials [24]. Carbon nanotubes, Au nanorods and Ag nanoparticles have been also successfully transferred from water to organic solvents and transferred back to water, by using PILs as vehicles [25]. In addition, optically active nanocomposites based on PILs and nanostructured metals or semiconductors have been prepared for plasmonics, optical applications and for fuel cell production [20,22,26,27]. Current efforts focus on the *in situ* polymerization of IL monomers in the presence of nanoparticles. However, the limited control on the preparative conditions represents the main drawback of such an approach to nanocomposite preparation, because it can detrimentally influence the properties of the final material [28,29]. Moreover, the relatively weak interactions between the *in situ* formed nanostructures and the surroundings may reduce the host matrix ability to coordinate the NC surface. Nanocomposites can be achieved also by *ex situ* methods, which involve the incorporation of pre-synthesized nano-objects in monomers before polymerization or, alternatively, directly in the host matrix. Thus, the *ex situ* approach allows for the direct transfer of the original size- and shape-dependent properties of colloidal NCs in the host matrix for finally obtaining a nanocomposite with defined and enhanced characteristics. In addition, the opportunity of tuning the inorganic NC surface chemistry and/or the host matrix specific composition allows for the improvement of the affinity degree between the two components, thanks to an effective passivation at the NC surface.

In this work, we report the preparation of novel hybrid nanocomposites, obtained by an *ex situ* approach, formed by pre-synthesized colloidal CdSe NCs and imidazolium-based PILs, namely, poly(*N*-vinyl-3-butylimidazolium hexafluorophosphate) (PVBuIm(PF_6_)) or the homologue PIL functionalized with a thiol end-group, SH-PVBuIm(PF_6_) (SH-PIL in the following). Thiol functionalities, indeed, have been introduced on the PIL chain to study the effect of this moiety on the dispersion of the NCs in the polymer matrix and on the ultimate morphological and optical properties of the nanocomposite. A capping exchange procedure of the hexadecylamine (HDA) and trioctylphosphine oxide (TOPO)-capped CdSe NCs has been performed for replacing the organic ligands with inorganic S^2−^ ions and allowing the incorporation of the CdSe NCs in the PILs by means of a common polar solvent, namely dimethyl sulfoxide (DMSO). The as-prepared nanocomposites have been extensively studied by morphologic and spectroscopic measurements, by both time-integrated and time-resolved techniques, to evaluate possible interactions between the PILs and the NCs. Experimental results demonstrate that the surface functionalization of the CdSe NCs with S^2−^ ions results in a dispersion of the nano-objects in the PILs, allowing for the achievement of stable and optically transparent nanocomposites. Upon incorporation, the NCs retain their size, shape and composition. In particular, the role of the functionalization of the polymeric chains in the modification of the optical properties of the nanocomposite has been highlighted, evidencing that thiol groups provide favorable interactions with the NC surface, which preserve the optical properties of the nano-objects. Moreover, spectroscopic results suggest the occurrence of charge transfers from the SH-PIL to the CdSe NC emitting states, thus demonstrating the effective interaction between the two components. The possibility of probing the interactions between NCs and PILs has resulted in a relevant contribution to enhance the knowledge in the field of innovative nanocomposite systems, as materials for potential integration in functional devices. Indeed, NC/PIL-based nanocomposites could be exploited as electrolytes in light-emitting electrochemical cells, combining inorganic luminescent semiconductors and solid polymeric electrolytes in a unique material. Therefore, the prepared nanocomposites, formed by inorganic semiconducting NCs and PILs, represent the starting point for the development of innovative electroactive nanocomposite materials for the fabrication of electrochemical and electrochromic devices and actuators.

## 2. Results and Discussion

**Scheme 1.**
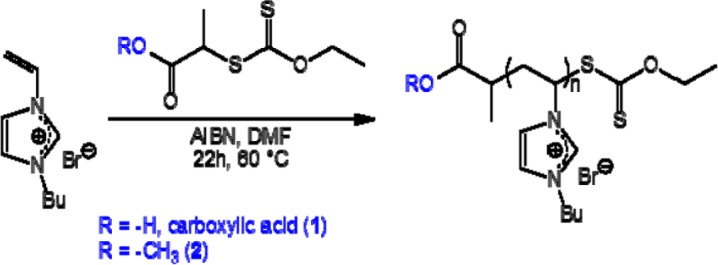
Synthesis of poly(*N*-vinyl-3-butylimidazolium bromide (Br) (PVBuImBr) by reversible addition fragmentation chain transfer (RAFT) polymerization.

**Scheme 2.**
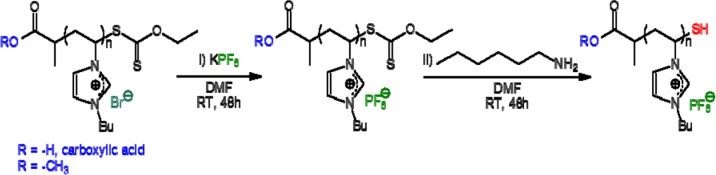
Anion exchange reaction, from bromide to hexafluorophosphate (PF_6_), to obtain PVBuIm(PF_6_), followed by aminolysis, leading to the thiol-functionalized PVBuIm(PF_6_).

PVBuIm(PF_6_) (PIL in the following) has been obtained by simple anion exchange at room temperature, from the bromide (Br^−^) of a PVBuImBr precursor to hexafluorophosphate (PF_6_^−^). As for the SH functionalized PVBuIm(PF_6_) (SH-PIL in the following), it has been synthesized by chemical modification of the xanthate end-group into thiol [30]. Namely, the synthesis of the PVBuImBr precursor has been achieved by reversible addition fragmentation chain transfer (RAFT) polymerization, at 60 °C, in dry dimethylformamide (DMF) using xanthate **1** or **2** as a chain-transfer agent (CTA) and Azobis(2-methylpropionitrile) (AIBN) as the radical source ([M]/[AIBN]/[CTA] = 34/0.2/1) (see Scheme 1). The RAFT process thus introduced a xanthate end-group in the omega position of the PVBuImBr chain (theoretical molecular weight, ( = 6500 g/mol). The reaction has been quenched by cooling, and the solution has been poured in ether/acetone (1/1) to remove residual monomers. Conversion has been calculated by comparing the signals due to the vinylic protons of the monomers at 5.4–6 ppm with protons –CH_3_ of the butyl substituent (0.8 ppm). PVBuIm(PF_6_) has been next obtained by simple anion exchange at room temperature, from bromide to hexafluorophosphate, using DMF as the solvent (see Scheme 2). Finally, chemical modification of the xanthate end-group into thiol has been achieved by aminolysis of PVBuIm(PF_6_) [30], as depicted in Scheme 2.

Luminescent HDA/TOPO-capped CdSe NCs have been synthesized by the colloidal route reported by Mekis *et al*. [31]. A capping exchange procedure has then been performed in order to replace the pristine HDA and TOPO capping molecules from the surface of the CdSe NCs with ionic S^2−^ ion ligands, by treatment with ammonium sulfide (NH_4_)_2_S. This has permitted the dispersal of the nano-objects in the PILs by using DMSO as the polar common solvent. Indeed, PILs are typically soluble in polar solvents, which are not compatible with the surface chemistry of the nano-objects derived directly from the synthetic procedure.

**Figure 1.**
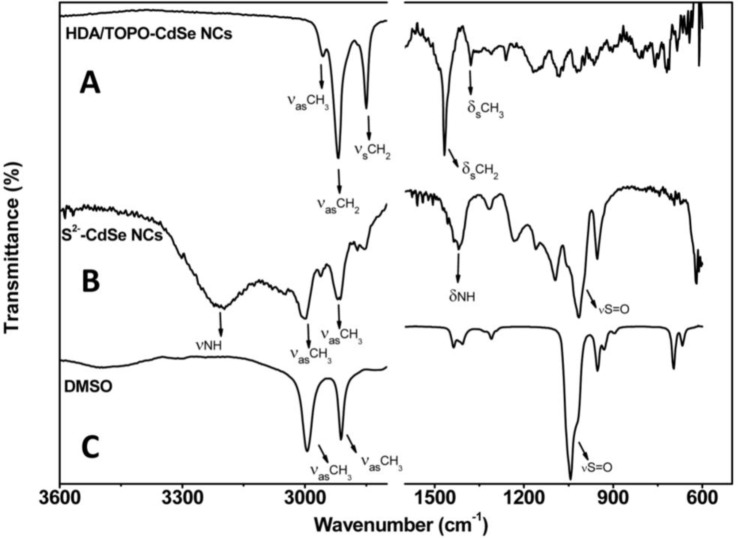
Attenuated total reflection Fourier transform infrared (ATR-FTIR )absorption spectra of (**A**) CdSe NCs coated by trioctylphosphine oxide (TOPO) and hexadecylamine (HDA) in chloroform; (**B**) S^2−^ stabilized CdSe nanocrystals (NCs) in dimethyl sulfoxide (DMSO) and (**C**) pure DMSO.

The surface of the CdSe NCs, before and after treatment with (NH_4_)_2_S, has been studied by ATR-FTIR infrared spectroscopy in order to verify the effective accomplishment of the exchange of pristine HDA/TOPO ligands with S^2−^ ions. Figure 1 compares spectra of the pristine HDA/TOPO-capped CdSe NCs, of the S^2−^ stabilized CdSe NCs and of DMSO.

The narrow bands at 2956, 2917 and 2850 cm^−1^ can be ascribed to the –CH_3_ asymmetric stretching and to the –CH_2_ asymmetric and symmetric stretching, respectively, of the alkyl chains of TOPO and HDA molecules. At lower wavenumbers, the narrow bands at 1467 and 1378 cm^−1^ are ascribed to bending vibrations of C–H in the –CH_2_ and –CH_3_ moieties, respectively.

The IR spectrum of the NCs stabilized by the S^2−^ ions shows two bands at 3197 and 1419 cm^−1^, respectively, evidencing the presence of ammonium ion, which contributes as the counter ion in stabilizing the NCs in DMSO. In particular, the broad and strong band pointing at 3197 cm^−1^ is due to N–H stretching vibrations, while the broad signal at 1419 cm^−1^ can be ascribed to the N–H bending. Two distinct strong bands at 2994 and 2911 cm^−1^ are instead typical of both the asymmetric and symmetric stretching of the –CH_3_ groups of DMSO, whose spectrum is also shown in the same figure for comparison. The strong vibration at 1014 cm^−1^ is still accounted for as the S=O stretching of DMSO. The weak signals at 2998, 2916 and 2856 cm^−1^, at 2871 cm^−1^ and those at lower wavenumbers, lacking in the spectrum of pure DMSO, can be accounted for as the pristine TOPO and HDA ligands almost completely removed in the capping exchange.

After capping exchange, the solution of the NCs was optically transparent and was found stable in DMSO thanks to the binding of the S^2−^ ions to the Cd^2+^ sites and the electrostatic repulsion among the charged NCs, preventing their aggregation.

**Figure 2.**
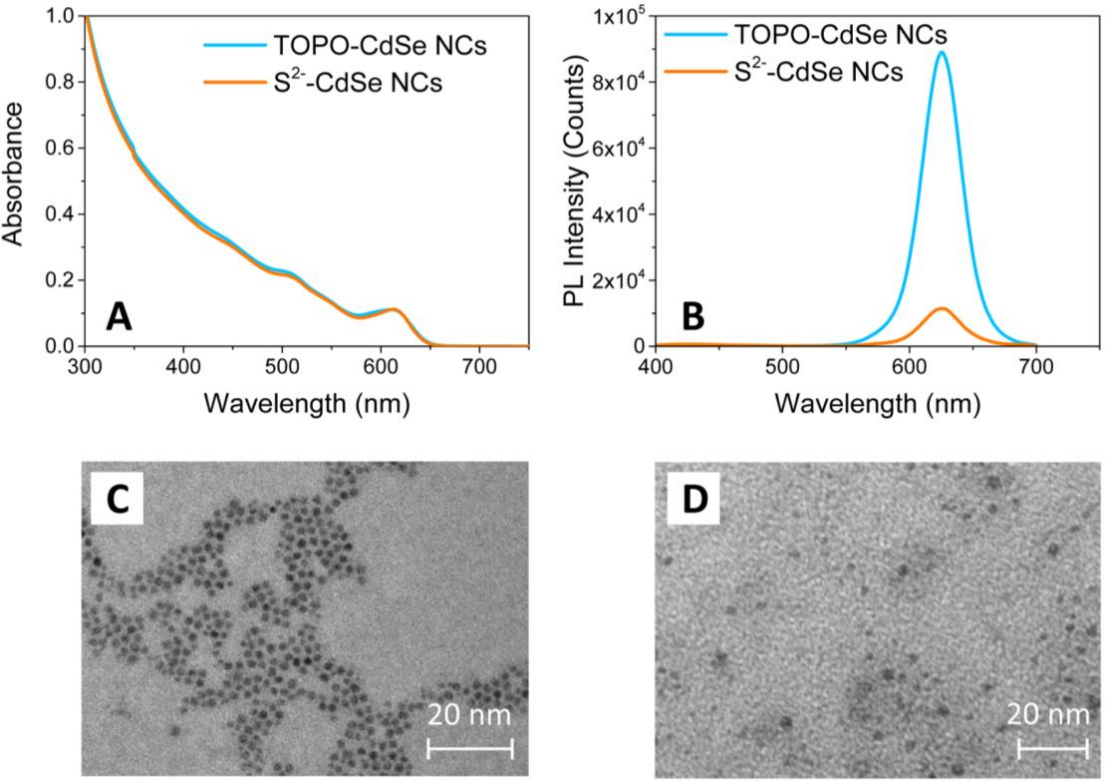
(**A**) UV-Vis absorption and (**B**) PL emission spectra (λ_Exc_ = 375 nm) of TOPO-capped and S^2−^-capped CdSe NCs CHCl_3_ and DMSO solution, respectively. TEM images of (**C**) pristine TOPO-capped CdSe NCs and (**D**) S^2−^-capped CdSe NCs.

Characterization by TEM and UV-Vis spectroscopy of the CdSe NCs has been performed in order to evaluate the preservation of the morphological and optical properties of colloidal nanostructures after the capping exchange. Figure 2 shows the comparison between the UV-Vis absorption (Panel A), photoluminescence (PL) emission spectra (Panel B) and TEM images (Panels C and D) of the HDA/TOPO-capped and of the S^2−^-stabilized CdSe NCs.

The characteristic absorption spectrum of the as-synthesized HDA/TOPO-capped CdSe NCs dispersed in CHCl_3_ (light blue line of Figure 1A) shows a main excitation peak attributed to the first optically allowed transition. The average NC diameter has been obtained from the wavelength position of the absorption edge and is found around 5.7 nm [32], in agreement with the size estimated by TEM images (Figure 2C). After capping exchange, the UV-Vis absorption spectrum of the S^2−^ stabilized CdSe NCs dispersed in DMSO is proven to fully superimpose to that of the pristine colloidal NCs (orange line in Figure 2A). The position of the exciton peak does not change, evidencing a retention of the size and shape of the NCs after processing with the (NH_4_)_2_S solution. As a confirmation, the TEM micrograph of the S^2−^ stabilized CdSe NCs shows spherical nanoparticles of *ca.* 5–6 nm in size (Figure 2D).

Figure 2B displays the PL emission spectra of the CdSe NCs before and after capping exchange. The CdSe NCs show an intense narrow band-edge emission in CHCl_3_, revealing a fine NC size distribution. After processing with (NH_4_)_2_S, the maximum of the band-edge PL emission of the S^2−^-stabilized CdSe NCs in DMSO is not shifted, confirming that the capping exchange does not modify the geometrical properties of the NCs. On the other hand, the figure shows a significant decrease of the PL intensity of the S^2−^ stabilized CdSe NCs in DMSO. Such a quenching can be ascribed to some desorption effects of the organic ligands, which provide the occurrence of defect sites on the NC surface. Such defect states are typically localized within the energy gap and effectively compete with the PL emission [33]. However, in spite of such a decrease in PL intensity, the main optical and morphological properties of the colloidal CdSe NCs are retained after processing with ammonium sulfide. Therefore, the CdSe NCs stabilized by the S^2−^ ions can be successfully dispersed in a polar solvent, such as DMSO or formamide, for preparing optically transparent and stable solutions.

The possibility of transferring the colloidal NCs in a polar host environment allows for further post-synthetic processing, extending the applicability of the nano-objects for diverse purposes. In particular, here, the S^2−^-stabilized CdSe NCs have been dispersed in DMSO, with a fixed concentration of 10^−5^ M, and then incorporated in the PILs (0.1 g), to obtain stable and novel organic-inorganic nanocomposite materials.

PVBuIm(PF_6_) and its thiol end-functionalized homologue have been used as host matrices for the S^2−^-stabilized CdSe NCs. Thiol groups are expected to coordinate the NC surface, partially replacing the pristine ligands, thus increasing the coordination degree of the NC surface and avoiding the aggregation of the nano-objects [34].

The nanocomposites have been analyzed by spectroscopic and morphological techniques to study the interactions between the PILs and the NCs, the influence of the functionalization of the PIL on the distribution of the NCs in the matrix, as well as the optical properties of the nanocomposite material.

**Figure 3.**
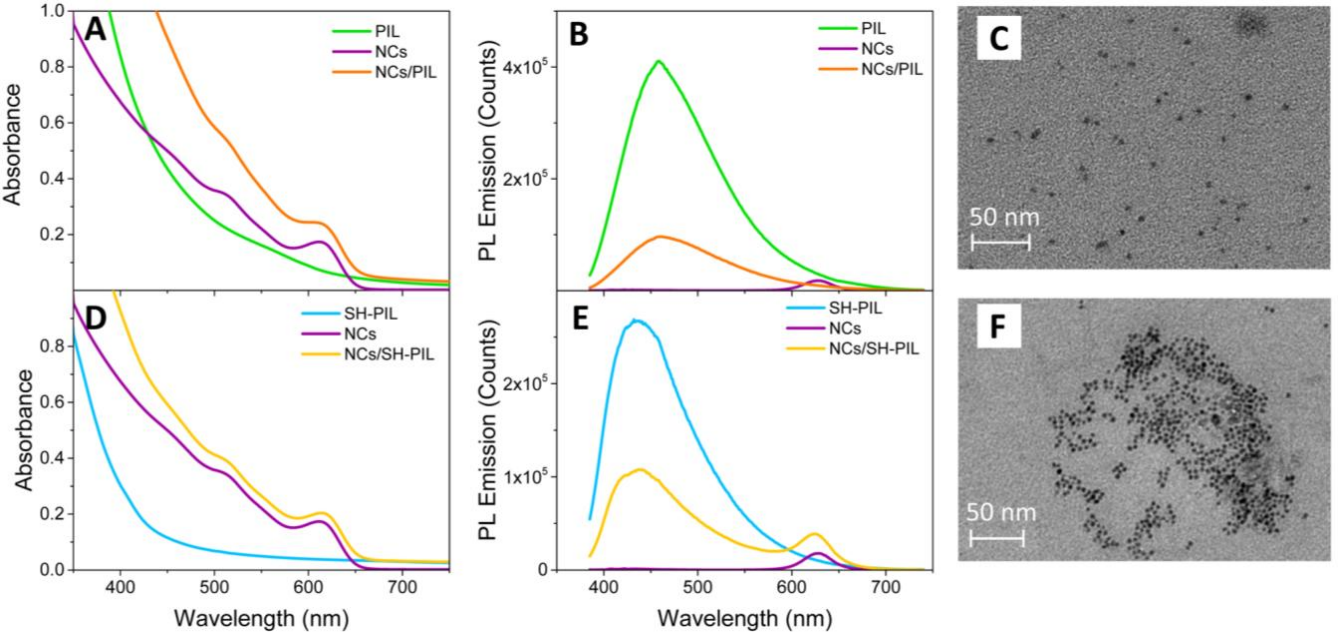
(**A**) UV-Vis absorption and (**B**) PL emission spectra (λ_Exc_ = 375 nm) of polymeric ionic liquid (PIL) -based nanocomposite and the bare components in DMSO dispersion; (**D**) UV-Vis absorption and (**E**) PL emission spectra (λ_Exc_ = 375 nm) of SH-PIL-based nanocomposite and the bare components. TEM images of (**C**) NC/PIL and (**F**) NC/SH-PIL nanocomposites deposited onto a copper-coated grid by spin-coating at 6000 rpm from the corresponding DMSO dispersion.

Figure 3 shows the comparison among the UV-Vis absorption (Panel A) and PL emission (Panel B) spectra of the CdSe NCs/PIL nanocomposites and of both the bare components. UV-Vis absorption (Panel D) and PL emission (Panel E) spectra of the SH-PIL-based nanocomposites and of the corresponding bare components are also provided.

As shown in Figure 3A, the CdSe NCs/PIL nanocomposite in DMSO results in an optically clear dispersion, the absorption spectrum of which attests to the presence of both the inorganic NCs and the PIL components. In the nanocomposite absorption spectrum of Figure 3A, the characteristic excitonic absorption peak is indeed ascribable to the CdSe colloidal NCs, an intense absorbance band below 400 nm being observed. In the UV spectral region, both the colloidal NCs and the imidazolium-based PILs absorb. The absorption of the CdSe NCs is due to the continuum of the states typical of semiconductors, while that of the PIL solution in DMSO is ascribed to π–π* transitions of the imidazolium ring [35,36]. The tail, which instead appears in the spectra of the PIL in the visible range, can be ascribed to the presence of energetically different associated structures, originated from strong interactions among the π delocalized orbitals of the imidazolium ring [35,36]. Nevertheless, due to the high absorption between 500 and 650 nm, the presence of some residual impurities originating from the synthetic procedure of the IL and/or from halide ions preceding the anion exchange in the PIL synthesis cannot be ruled out [37,38].

Figure 3B shows the PL emission spectrum of the NC/PIL nanocomposite, compared with those of the bare CdSe NCs and the PIL in DMSO. Although the absorption features of both components are retained in the nanocomposite dispersion, only the PIL characteristic emission is observed in the PL emission spectrum of the nanocomposite, even if a decrease in the emission intensity is detected (Figure 3B). Indeed, the PL emission spectra of the nanocomposite shows a typical wide fluorescence band centered on 458 nm, which is ascribable to the fluorescence of the imidazolium associated structures, whose tail could hide the band-edge PL emission of the CdSe NCs.

The SH-PIL-based nanocomposite also results in an optically clear dispersion in DMSO. The related UV-Vis absorption spectrum shows characteristic peaks of both the NCs and the thiol functionalized PIL (Figure 3D). In particular, the spectrum of the nanocomposite reflects the typical line-shape of the CdSe colloidal NCs in which the first allowed excitonic transition is visible at 614 nm, while the increase of the absorption intensity below 450 nm is due to the contribution of the SH-PIL. The thiol functionalized PIL shows a strong absorption band in the UV region of the spectrum and a long tail extended up to 500 nm, which is fully compatible with the absorption features of the associated structures formed by the stacking of the imidazolium rings, as observed and discussed in recently published papers [35,36].

Figure 3E shows the PL emission spectra of the NC/SH-PIL nanocomposite and of the bare components. The SH-PIL-based nanocomposite exhibits a wide fluorescence band centered on 436 nm, ascribable to the fluorescence of the PIL and typically attributed to the PL emission of imidazolium ring-associated structures. Conversely to what is observed in the case of the NC/PIL nanocomposite, the weak band-edge emission of the CdSe NCs is still observed at 614 nm in the case of the PIL bearing the thiol end-group. Hence, the SH groups favorably interact with the CdSe NC surface, preserving the band-edge PL. Such a behavior suggests that the thiol functionality plays an important role in promoting mutual interactions between PIL and NCs at their interface, improving the optical properties of the nanocomposite [39].

TEM analyses have been also carried out to investigate the morphological properties of the CdSe NC/PIL and CdSe NC/SH-PIL nanocomposites. Figure 3C,E show nanostructures having a size compatible with that of the CdSe NCs and which are homogeneously dispersed in the polymeric hosts. This result evidences that the S^2−^ inorganic ligand can stabilize the nano-objects in the PIL matrix.

In summary, steady-state UV-Vis spectroscopy and TEM measurements demonstrate a complete retention of the size, shape and size-distribution of the S^2−^-stabilized CdSe NCs upon their incorporation in both the PILs, with no phase separation or aggregation of the colloidal nanostructures occurring within the host matrix. In addition, a quenching of the CdSe NC band-edge emission has been observed in the case of the non-functionalized PIL-based nanocomposite.

TR-PL experiments have been then performed in order to get a deeper insight in to the possible interactions, such as charge or energy transfers, between the organic and inorganic components of the nanocomposites.

**Figure 4.**
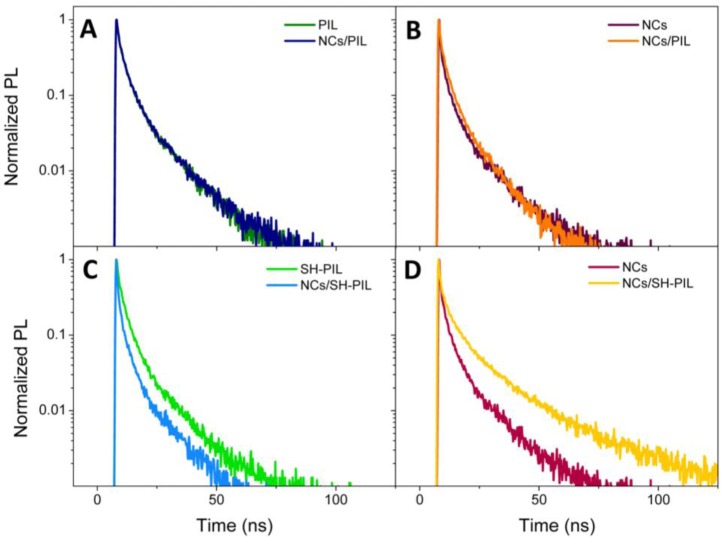
Normalized TR-PL emission decay of the NCs/PIL nanocomposite DMSO dispersion compared with the bare components, at the (**A**) PIL and (**B**) NC maximum emission wavelengths. The normalized TR-PL emission decay of the NCs/SH-PIL nanocomposite DMSO dispersion compared with the bare components, at the (**C**) SH-PIL and (**D**) NC maximum emission wavelengths.

Figure 4 reports normalized PL decays of nanocomposite DMSO dispersions, compared to those of the bare components, in correspondence with the PIL and CdSe NC fluorescence peak. In particular, Panel A shows the PL decays of the PIL and the CdSe NC/PIL DMSO dispersion detected at 458 nm, which is the wavelength of maximum intensity of PIL fluorescence (see Figure 3B). Figure 4B reports the comparison between similar decays of NC/PIL nanocomposite and the S^2−^-capped CdSe NCs, revealed at the NC band-edge peak emission, namely 626 nm (see Figure 3B). Analogously, Figure 4C reports the comparison between PL decays of the SH-PIL and of the corresponding nanocomposite DMSO dispersion, detected in correspondence with the SH-PIL PL maximum intensity (436 nm) (see Figure 3E). Instead, Figure 4D shows the comparison between PL decays of the CdSe NCs and of the SH-PIL based nanocomposite, revealed at the CdSe NC band-edge PL maximum emission (626 nm) (see Figure 3E).

The PL decays of both PILs, CdSe NCs and nanocomposites exhibit a non-mono-exponential trend, consistent with that reported in the literature [36,40,41]. To obtain the characteristic lifetimes of the emitting states, experimental decays have been best fitted by a tri-exponential function, in accordance with previous reports regarding CdSe NCs and imidazolium-based ILs [36,40,41]. Recent studies have demonstrated that the multiple exponential decays can be effectively represented by average lifetimes, which can well describe complex systems, where different excited states participate in recombination dynamics [42,43]. Average PL lifetimes (τ_Avg_), which are the average time after the excitation pulse at which an emitted photon is detected, have been calculated according to Jones, M. and Scholes, G. D. [42,43], on the basis of the three time component values and their associated amplitudes. The average PL lifetimes have been calculated by Equation (1) and are reported in Table 1:

$$\tau_{\text{Avg}}=\sum_{n}\frac{A_{n}\tau_{n}^{2}}{\Sigma {}_{m}A_{m}\tau_{m}}$$ (1)

TR-PL measurements performed on the NC/PIL nanocomposite DMSO dispersion reveal a substantial overlapping of the PL decays observed at the maximum emission wavelength of the PIL (Figure 4A) and of the NCs (Figure 4B). As reported in Table 1, the calculated τ_Avg_ is ~11 ns in the PIL emission region, either in absence or in presence of the CdSe NCs. On the contrary, in correspondence with the NC band-edge emission, τ_Avg_ passes from 13.2 ns for the CdSe NC DMSO dispersion to 12.7 ns for the nanocomposite NC/PIL. Such a slight difference in the calculated average lifetimes, although not visibly observed in the PL decays of Figure 4B, can be indicative of a partial quenching of PL band-edge emission occurring after the incorporation of the S^2−^-stabilized CdSe NCs in the PIL. In fact, quenching of the semiconducting NC band-edge fluorescence has been observed in the PL emission spectrum of the NC/PIL nanocomposite (Figure 3B). However, a very weak residual fluorescence could be hidden by the emission tail of the PIL.

TR-PL experiments performed on the SH-PIL based nanocomposite show a different behavior. Indeed, TR decays observed at the maximum emission wavelength of both the SH-PIL (436 nm) and the NCs (626 nm; see Panel C and Panel D of Figure 4, respectively) significantly differ. In the SH-PIL emission range, the SH-PL decay becomes faster in the presence of colloidal NCs (Figure 4C). The calculated τ_Avg_ decreases from 11.2 ns for the bare SH-PIL to 9.6 ns in the nanocomposite DMSO dispersion. Conversely, at the CdSe NC maximum emission wavelength, the SH-PL decay rate of the nanocomposite undergoes a considerable slowing-down. Namely, the τ_Avg_ calculated from decays detected at 626 nm increases from 13 ns for the bare CdSe NCs up to 26 ns for the nanocomposite dispersion.

Typically, TR-PL experiments can exploit the high sensitivity of the NC luminescence to surface states and chemical environments for providing information on the emitting state dynamics that are involved between NC charge carriers and the surrounding environment, such as energy or charge transfer phenomena. Indeed, possible direct interactions between NCs and PIL could modify the surface states of NCs and, therefore, affect the PL decays. A charge transfer mechanism could be responsible for the observed increase of lifetime in the NC emission spectral region and the concomitant faster decay in correspondence with the SH-PIL spectral emission wavelength. In the excitation region (375 nm), both NCs and SH-PIL have a quite intense absorbance. Then, the excitation photons can be easily absorbed by both the components, and excited electrons of the imidazolium ring-associated structures of the SH-PIL could be transferred to the CdSe NCs, which emit from lower energy states. Such a possible charge transfer could contribute to the deactivation processes of the SH-PIL emitting states, resulting in a faster decay of the SH-PIL component and a concomitant increase of the NC lifetime. Such a hypothesis can be experimentally supported by the concomitant decrease in the PL lifetime of the SH-PIL in the presence of the CdSe NCs (Figure 4C), and the slowing-down of the NC PL decay in the nanocomposite DMSO dispersion (Figure 4D).

The TR-PL data evidence that the thiol functionality hanging from the PIL chain-ends can coordinate the NC surface and favor the electron coupling between the different components of the nanocomposite. These results highlight the potential of these novel hybrid materials for optoelectronic and energy conversion devices.

## 3. Experimental Section

### 3.1. Materials

All chemicals were of the highest available purity and used as received, without further purification. Cadmium oxide (CdO, powder, 99.5%), selenium (Se, powder, 99.99%), trioctylphosphine oxide (TOPO, technical grade), tributylphosphine (TBP, 99%), tert-butyl phosphonic acid (TBPA) dimethyl sulfoxide (DMSO, 99.7%), ammonium sulfide solution ((NH_4_)_2_S, 40–48 wt% in water) and hexane (99%) were purchased from Aldrich. Hexadecylamine (HDA, 99%) was purchased from Fluka.

Azobis(2-methylpropionitrile) (AIBN, 99%) was received from Aldrich and purified by recrystallization from methanol. N-vinylimidazole (99%) and 1-bromobutane (99%) were obtained from Alfa Aesar and used as received. Potassium hexafluorophosphate (KPF6, >98%) was received from Roth and used as received. *O*-Ethyl-S-[(1-methoxycarbonyl)ethyl] dithiocarbonate (**1** in Scheme 1) and *O*-ethyl-S-[(1-methoxycarbonyl)ethyl] dithiocarbonate (**2** in Scheme 1) were prepared following a procedure already described [44,45]. Hexylamine (99%, Sigma-Aldrich, Seelze, Germany), dimethylformamide (DMF) (≥99.7%, Scharlau, Barcelona, Spain), tetrahydrofuran (THF) (99.5%, Fischer, Germany), acetone (99.5%, VWR, Fontenay-sous-Bois, France), diethyl ether (anhydrous, Baker, Deventer, Netherlands) and pentane (>99.5%, Fluka) were used without further purification.

### 3.2. Synthesis of PILs

#### 3.2.1. Synthesis of Poly(*N*-vinyl-3-butylimidazolium hexafluorophosphate) (PVBuIm(PF_6_))

Poly(*N*-vinyl-3-butylimidazolium hexafluorophosphate) (PVBuIm(PF_6_)) was synthesized by anion exchange from poly(1-vinyl-3-butylimidazolium) bromide (PVBuImBr). This precursor was synthesized from *N*-vinyl-3-butylimidazolium (VBuImBr) by RAFT polymerization.

*N*-vinyl-3-butylimidazolium (VBuImBr) bromide was prepared by quaternization following a procedure already described [46–49]. The monomer was recovered as a white solid with 100% yield and investigated by NMR spectroscopy, whose data perfectly matched those reported in the literature. In a typical experiment, a 10 mL Schlenk tube was flame dried and charged with [VBuImBr]/[AIBN]/[CTA] = 34/0.2/1 in 10 mL of dry DMF. The Schlenk tube was subjected to five freeze-thaw cycles and placed in a thermostated oil bath previously maintained at 80 °C. The reaction was quenched after 24 h by sudden cooling with liquid nitrogen. The resulting PVBuImBr was isolated by precipitation in acetone/diethyl ether (1/1). After drying under vacuum, PVBuImBr was obtained as a white powder (yield 83%–85%). An aliquot was taken to determine the conversion by ^1^H NMR in DMSO-d6 (85% conversion, = 6500 g/mol). ^1^H NMR (CD_3_OD): δ 7.3–7.8 (br, CH=CH, 2H), 4.4–4.8 (br, N–CH–CH_2_, 1H), 3.9–4.5 (br, N–CH_2_–CH_2_–CH_2_–CH_3_, 2H), 2.4–3.0 (br, N–CH–CH_2_, 2H), 1.7–2.1 (br, CH_3_–CH_2_–CH_2_–CH_2_–, 2H), 1.3–1.6 (br, CH3–CH_2_–CH_2_–CH_2_–, 2H), 0.9–1.2 (br, CH_3_–CH_2_–CH_2_–CH_2_–, 3H). NMR data were in agreement with those reported in the literature [46].

PVBuIm(PF_6_) was achieved by anion exchange from PVBuImBr. This metathesis reaction was performed following a procedure already described in the literature [46,47,50]. Three grams (12.9 mmol) of PVBuImBr were first dissolved in 5 mL of DMF. The resulting solution was added to a stirred solution of potassium hexafluorophosphate (KPF_6_ (2.61 g, 1.1 eq., 14.2 mmol)), previously dissolved in 5 mL of DMF, for 48 h. The mixture was poured in 200 mL of water and washed a few times. The corresponding PVBuIm(PF_6_) was removed by filtration and dried under vacuum at 40 °C overnight (80% yield). The PVBuIm(PF_6_) was tested with a solution of AgNO_3_ to check the completion of anion exchange.

#### 3.2.2. Synthesis of SH End-Functionalized PVBuIm(PF_6_) *via* Aminolysis

Three grams (10.2 mmol) of PVBuIm(PF6) were dissolved in DMF (5 mL), and the solution was purged with nitrogen for 15 min. A solution of 43 mg (0.05 eq., 0.5 mmol) of hexylamine, previously dissolved in DMF (5 mL) and purged, was added under nitrogen. After stirring for 48 h at room temperature, the polymer was purified by two consecutive precipitations in pentane. A white-yellowish powder was obtained and dried under vacuum at 40 °C overnight (85% yield).

### 3.3. Synthesis of Colloidal CdSe NCs

#### 3.3.1. Synthesis of HDA/TOPO-Capped CdSe NCs

CdSe colloidal NCs were prepared by adapting a previously reported synthetic procedure [31]. All the synthetic steps were performed under nitrogen atmosphere by using the standard air-free technique starting from dried and degassed reactants. Briefly, in a typical synthesis, CdO powder was dissolved in a mixture of TOPO, HDA and TBPA surfactants and degassed at 120 °C. After CdO dissolution, the mixture was heated up to 290 °C. At this temperature, TBP was injected into the mixture. Then, the temperature was further raised up to 300 °C, and a TBP solution of Se precursor was quickly injected into the hot reaction solution. After such an injection, the temperature was decreased down to 270 °C, allowing NC growth for a time, dependent on the desired NC size. After an annealing step at 110 °C, the as-prepared CdSe NCs were carefully washed and purified by a solvent non-solvent precipitation with methanol, which allowed for the removal of the excess of ligands and the non-reacted precursors. Finally, the purified NCs were dispersed in chloroform for obtaining optically transparent solutions. The molar concentration of the CdSe NC chloroform solution, as well as the average size of the NCs were estimated as reported by Peng *et al.* [32].

#### 3.3.2. Preparation of S^2−^-Capped CdSe NCs

The capping exchange procedure was performed by following a recent method reported by Talapin *et al*. [4]. Such a procedure, which uses a metal-free inorganic ion, such as ammonium sulfide ((NH_4_)_2_S), has been applied, herein, for replacing the pristine layer of organic capping ligands, namely HDA and TOPO, on the surface of the CdSe NCs.

The “as-synthesized” CdSe NCs were processed with ammonium sulfide (NH_4_)_2_S in air, as well as under inert atmosphere, and stable colloidal dispersions of CdSe NCs have been prepared in DMSO. In particular, 1 mL of colloidal CdSe NC solution (~10^−4^ M) in either toluene or hexane was mixed to a solution of (NH_4_)_2_S in DMSO (Figure 1). The last was prepared by adding 10 μL of the (NH_4_)_2_S solution to 1 mL of DMSO. The mixture was stirred for about 10 min, leading to a complete phase transfer of CdSe NCs from toluene or hexane to the DMSO phase. The phase transfer can be easily monitored by a color change of the toluene or hexane (red to colorless) and of the DMSO (colorless to red) phases. The DMSO phase was separated out, followed by triple washing with toluene to remove any remaining nonpolar organic species. The washed DMSO phase was then filtered through a 0.2 μm polytetrafluoroethylene (PTFE) filter, and ~1 mL of acetonitrile was added to precipitate out the NCs. Finally, the sulfur-stabilized CdSe NCs were dispersed in DMSO for achieving stable solutions with a concentration of about 10^−5^ M.

### 3.4. Preparation of S^2−^-Capped CdSe NC/PIL Nanocomposites

Imidazolium-based PILs, namely PVBuIm(PF_6_) (PIL in the following) and SH-functionalized PVBuIm(PF_6_) (SH-PIL in the following), were used to prepare nanocomposites with S^2−^-stabilized CdSe NCs. Preliminary tests of the PIL solubility in several solvents have been performed. The PILs are soluble in DMSO, DMF and acetonitrile (ACN) while only at a low concentration in the solvents typically used for dispersing the as-synthesized colloidal CdSe NCs. Therefore, in order to obtain CdSe NCs dispersible in polar organic solvents, principally DMSO, it was necessary to modify the NC surface chemistry by means of a capping exchange procedure. Both the nanocomposites, NC/PIL and NC/SH-PIL, were prepared by adding 1 mL of a S^2−^-capped CdSe NC DMSO solution (~10^−5^ M) to 0.1 g of PIL (or SH-PIL). The mixture was then stirred for about 30 min, until the PIL (or SH-PIL) was completely dissolved in the NC DMSO dispersion and an optically clear solution was achieved. Both the nanocomposites preserved their optical features for about 1 month in DMSO.

### 3.5. NMR Analyses

^1^H NMR and ^13^C NMR spectra were recorded on a Bruker AC-400 spectrometer in appropriate deuterated solvents.

### 3.6. Spectroscopic Investigation

Attenuated total reflection Fourier transform infrared (ATR-FTIR) spectra were recorded by means of a PerkinElmer Spectrum One Fourier Transform Infrared spectrometer equipped with both a deuterated triglycine sulfate detector and a three-bounce, 4-mm diameter diamond microprism as the internal reflection element. The resolution was 4 cm^−1^. The measurements were performed by dropping aliquots (3–5 μL) of the chloroform solutions of the CdSe NCs directly onto the upper face of the diamond crystal, and the spectra were acquired upon solvent evaporation.

UV-Vis absorption spectra were recorded with a Cary 5000 (Varian) UV/Vis/NIR spectrophotometer (Agilent Technologies, Santa Clara, CA, USA). PL spectra were recorded by using a Fluorolog 3 spectrofluorometer (HORIBA Scientific, Jobin-Yvon, Edison, NJ, USA), equipped with double grating excitation and emission monochromators. All optical measurements were performed at room temperature on samples obtained directly from synthesis without any size sorting treatment. TR-PL measurements were performed by the time-correlated single photon counting (TCSPC) technique, with a FluoroHub (HORIBA Scientific, Jobin-Yvon). The samples were excited at 375 nm by a picosecond laser diode (NanoLED 375L, HORIBA Scientific, Jobin-Yvon) emitting τ ≈ 80 ps pulses at a 1-MHz repetition rate. The PL signals were dispersed by a double grating monochromator and detected by a picosecond photon counter (TBX ps Photon Detection Module, HORIBA Scientific, Jobin-Yvon). The temporal resolution of the experimental set up was ~200 ps.

### 3.7. Morphological Investigation

TEM analysis was performed using a JEM-1011 microscope (JEOL USA, Inc., Peabody, MA, USA), working at an accelerating voltage of 100 kV. TEM images were acquired by a Quemesa CCD 11 Mp Camera (Olympus, Münster, Germany). The samples were prepared by depositing ~10 μL of a diluted solution onto 400-mesh carbon-coated copper grids by spin coating at 6000 rpm for 1 min, to let the solvent evaporate. Size statistical analysis (NC average size) of the samples were performed by the freeware ImageJ analysis program.

## 4. Conclusions

Synthesis of novel and stable hybrid nanocomposite materials has been achieved, by incorporating luminescent semiconducting CdSe NCs in a polymeric ionic liquid host. Luminescent CdSe NCs, synthesized by a colloidal route, have been included in imidazolium-based PILs, properly functionalized with a thiol end-group on each chain, with the view of developing a higher affinity towards the CdSe NC surface.

We have exploited the versatility of the colloidal NC surface chemistry and, in particular, the effective ability to be modulated by means of organic chemical routes, for incorporating colloidal NCs in a variety of chemical environments. In this work, a capping exchange procedure has been implemented at the NC surface, so as to replace organic pristine HDA/TOPO ligands with S^2−^ inorganic ions and, thus, incorporate the nano-objects in the PILs using DMSO as the common polar solvent.

Two distinct PILs have been considered, namely poly(*N*-vinyl-3-butylimidazolium hexafluorophosphate) and the thiol end-functionalized homologue. Investigation into the morphology of the nanocomposites show that the S^2−^ stabilized CdSe NCs preserve their size and shape upon incorporation in the PIL host and are homogeneously dispersed, without evidence of phase segregation and aggregation phenomena.

Spectroscopic measurements highlight the beneficial effect of the thiol functionality at the termini of PIL chains in completely retaining the optical properties of the CdSe NCs. In addition, the possible coordination of PIL functionalities to the NC surface favors the occurrence of charge transfer processes between the components, consistently from SH-PIL to the CdSe NCs emitting states.

The obtained results show the potential of the CdSe NC/SH-PIL-based hybrids and encourages the use of such a novel nanocomposite system for a potential integration in photovoltaic and optoelectronic devices. Indeed, the demonstrated preservation of the morphological and optical features of both components in the nanocomposite allows for the enriching of the technological fields, where PILs find applications with the peculiar size- and shape-dependent properties of inorganic nanostructures. Certainly, potential applications of PILs in the fabrication of electrochemical devices, such as lithium batteries, dye-sensitized solar cells, fuel cells, supercapacitors, light-emitting electrochemical cells and field effect transistors, could be extended to the class of obtained nanocomposites, formed by inorganic semiconducting NCs and PILs.

The reported results can represent the starting point for developing innovative electroactive nanocomposite hybrid materials for the fabrication of electrochemical and electrochromic devices and actuators. Further efforts can be addressed for applying the developed SH-PIL-based nanocomposites as innovative electrolytes in light-emitting electrochemical cells, which combine inorganic luminescent semiconductors and solid polymeric electrolytes in a single material.

## Figures and Tables

**Table 1.** Average decay times (τ_Avg_), calculated according to Jones and Scholes, by using lifetimes (τ*_i_*) and amplitudes (*A_i_*) obtained by a three-exponential fitting. Average lifetimes refer to the CdSe NCs, PIL and SH-PIL DMSO dispersion and their corresponding nanocomposite dispersions, measuring decays at the PIL PL peak (458 nm), at the SH-PIL peak (436 nm) and at the CdSe NC peak (626 nm) wavelengths.

Material	CdSe NC/PIL τ_Avg_ (ns)		CdSe NC/SH-PIL τ_Avg_ (ns)	
Wavelength	458 nm	626 nm	436 nm	626 nm
CdSe NCs	–	13.2 ± 0.2	–	13.2 ± 0.2
PIL or SH-PIL	11.3 ± 0.1	–	11.2 ± 0.1	–
Nanocomposite	11.6 ± 0.2	12.7 ± 0.1	9.6 ± 0.1	26.1 ± 0.5
